# Volatile organic compounds as a potential screening tool for neoplasm of the digestive system: a meta-analysis

**DOI:** 10.1038/s41598-021-02906-8

**Published:** 2021-12-09

**Authors:** Lixing Wang, Junan Li, Xiaoliang Xiong, Tingting Hao, Chao Zhang, Zhao Gao, Lili Zhong, Yinlong Zhao

**Affiliations:** 1 Department of Nuclear Medicine, The Second Hospital of Jilin University, Changchun, 130041 China; 2 Gastroenterology and Center of Digestive Endoscopy, The Second Hospital of Jilin University, Changchun, 130041 China; 3 Jilin Provincial Key Laboratory on Molecular and Chemical Genetic, The Second Hospital of Jilin University, Changchun, 130041 China

**Keywords:** Cancer screening, Gastrointestinal cancer, Tumour biomarkers

## Abstract

This meta-analysis was aimed to estimate the diagnostic performance of volatile organic compounds (VOCs) as a potential novel tool to screen for the neoplasm of the digestive system. An integrated literature search was performed by two independent investigators to identify all relevant studies investigating VOCs in diagnosing neoplasm of the digestive system from inception to 7th December 2020. STATA and Revman software were used for data analysis. The methodological quality of each study was assessed using the Quality Assessment of Diagnostic Accuracy Studies tool. A bivariate mixed model was used and meta-regression and subgroup analysis were performed to identify possible sources of heterogeneity. A total of 36 studies comprised of 1712 cases of neoplasm and 3215 controls were included in our meta-analysis. Bivariate analysis showed a pooled sensitivity of 0.87 (95% confidence interval (CI) 0.83–0.90), specificity of 0.86 (95% CI 0.82–0.89), a positive likelihood ratio of 6.18 (95% CI 4.68–8.17), and a negative likelihood ratio of 0.15 (95% CI 0.12–0.20). The diagnostic odds ratio and the area under the summary ROC curve for diagnosing neoplasm of the digestive system were 40.61 (95% CI 24.77–66.57) and 0.93 (95% CI 0.90–0.95), respectively. Our analyses revealed that VOCs analysis could be considered as a potential novel tool to screen for malignant diseases of the digestive system.

## Introduction

The incidence of the digestive system neoplasm is rising yearly worldwide. These tumors including colorectal cancer, stomach cancer, liver cancer, etc. have been associated high risk of morbidity and mortality based on the Global Cancer Statistics 2018^1^. Thus, early detection and timely treatment are integral for a favorable prognosis and long-term survival. At present, tumor markers have been widely used for tumor screening and diagnosis, monitoring treatment response, and surveillance of tumor recurrence after treatment. In clinical practice, markers commonly used to screen and diagnose tumors of the digestive system are mostly derived from blood, including carbohydrate antigen (CA50, 199, 242, 724), alpha-fetoprotein (AFP), carcinoembryonic antigen (CEA), pepsinogen, etc. However, the sensitivity and specificity of a single tumor marker are suboptimal, and it is often necessary to combine several tumor markers for the screening and early diagnosis of tumors^2–5^. Of course, there are also circulating tumor DNA and methylated genes from blood, urine or feces that are still being evaluated. Although these have shown good performance in the diagnosis of tumors of the digestive system, they have not been widely used in clinical practice due to immaturity in technology and high cost. Nevertheless, endoscopic examination with biopsy remains the gold standard for the diagnosis of gastric, esophageal, and colorectal cancers, while endoscopic ultrasonography and other imaging examinations can be used for the diagnosis of liver and pancreatic cancer. However, endoscopic screening and diagnostic approaches have limitations, including patient discomfort and potential major complications and harms. Therefore, an effective noninvasive screening tool for malignancies of the digestive system malignancies is desired.

Breath analysis is recognized as a simple and non-invasive method for screening and monitoring pathology or disease. Nevertheless, studies have shown that volatile organic compounds (VOCs) from urine also demonstrated good performance in the screening of cancer, in particular, special phenotypes of VOCs in urine could be used for the screening of prostate and gastrointestinal cancers^6^. Contrary to the conventional biomarker studies subjected to the health status of the individuals, the analysis of VOCs reflects the fingerprint characteristics of individuals that represent an instance of individualized medicine^7^. Moreover, a variety of sensor technologies are now being applied in analyzing the patterns of VOCs, such as eNoses and nanomaterials. As technology advances and clinical research progresses, VOCs analysis does not only enable early detection of cancer, but also the monitoring of the response to cancer therapy and detection of disease recurrence early when secondary treatments are most effective^8^.

VOCs are organic compounds with relatively low molecular weight and high vapor pressure. Cancer-originated VOCs have been frequently detected in feces, urine, blood, skin, sweat, and gases exhaled from cancer patients. These have been produced by tumor cells, which can reflect the disease^9–11^. Therefore, it is vital to explore the differences in VOCs released by varying cancer types to identify specific representative VOCs to each tumor that can be used as a diagnostic tool. For instance, the study by Kumar et al.^12^ has shown significantly lower concentrations of several specific volatile compounds in non-tumor individuals than those with tumors.

VOCs reflect changes in pathology and metabolic processes^13,14^. These specific VOCs have been considered to be the results of an imbalance between the systemic manifestations of oxidative stress, metabolic abnormalities, or reactive oxygen and the ability of the biological system to detoxify or repair damages^15–17^. In particular, the gut microbiome plays a key role in diseases of the digestive system, including colorectal cancer and inflammatory bowel disease. Changes in the composition of gut bacterial flora affect the fermentation products and the forms of volatile organic compounds (VOCs). Given that different diseases are associated with specific metabolomics that reflects cell metabolism, providing a new way of thinking for the diagnosis of diseases. In fact, in inflammatory bowel disease, changes in the gut microbiome are associated with colonic inflammation and can influence VOCs production. Also, some bacterial communities have been implicated in the development of colorectal cancer^18–21^. At the cellular level, changes in the production of VOCs have been associated with oxidation of polyunsaturated acids in the cell membrane as a result of genetic and/or protein mutations within tumor cells and the increased relative incidence of reactive oxygen species within cancer cells^22,23^. These cancer-related volatile organic compounds are released from the affected tissues into the feces or bloodstream and eventually excreted either through breath or through urine or feces^14^. Therefore, VOCs, as a comparatively new and non-invasive biomarker, provides a promising and attractive option for the screening of tumors in the digestive system.

Several studies have shown that VOCs analysis is valuable in the diagnosis of tumors of the digestive system^24–26^. However, these studies have some shortcomings, with some only analyzing VOCs in exhaled gas, while others had too a small sample size. In our meta-analysis, VOCs from all sources and tumors of the digestive tract were summarized and analyzed. This would ascertain the diagnostic efficacy of VOCs for tumors of the digestive system.

## Materials and methods

### Search strategy

A comprehensive, systematic electronic literature search of PubMed and Web of Science was conducted to identify all relevant papers from inception to 7^th^ December 2020. Search terms were as follows: volatile organic compounds or VOCs and esophageal neoplasm or esophageal cancer or gastric cancer or gastric neoplasm or liver cancer or liver neoplasm or hepatic neoplasm or pancreas cancer or pancreas neoplasm or colorectal cancer or colorectal neoplasm. All relevant literature was also screened for other possible studies.

### Study selections

All articles were reviewed by two independent investigators according to the inclusion and exclusion criteria. The inclusion criteria were: (1) VOCs were used to detect patients with neoplasm of the digestive system; (2) the control group consisted of patients with benign diseases of the digestive system and/or healthy individuals; (3) patients did not undergo any therapy; (4) extracted data could be used to measure true-positive (tp), false-positive (fp), false-negative (fn), and true-negative (tn) values. Studies/articles were excluded for the following criteria: (1) studies not within the field of our research; (2) non-human or animal study; (3) non-English literature; (4) non-diagnostic study; (5) studies on the neoplasm of not the digestive system; (6) data from the study were insufficient to constitute 2 × 2 tables; (7) review articles, editorials, case reports, conference proceedings, or letters.

### Data extraction

For each study included in our meta-analysis, the following data were extracted: first author, country, year of publication, number of participants, mean age of participants, percentage of males, cancer type, the source of VOCs, analytical platforms, and the control group. Also, each investigator registered and counted the numbers of tp, fp, tn, and fn. (Based on the criteria of diagnostic tests, we used the pathological results as the gold standard and then classified the results as true positive (tp), false positive (fp), true negative (tn) and false negative (fn) according to the degree of matching between the results obtained by pathology and VOCs analysis. Sensitivity was calculated as tp/(tp + fn), while specificity was calculated as tn/(tn + fp). Correspondingly, four basic data could be extracted from the known sensitivity and specificity. Any disagreements between the investigators were assessed by a third reviewer and resolved by mutual agreement.

### Quality assessment

To assess the quality of the included studies, the Quality Assessment of Diagnostic Accuracy Studies 2 (QUADAS-2) tool^27^ was used, which was an evidence-based quality assessment tool for systematic reviews of diagnostic accuracy studies that included four domains: patient selection, index test, reference standard, and flow and timing.

### Statistical analysis

Heterogeneities between the studies were evaluated by measuring the *P*-value of the Cochran-Q test and I-square statistic^28^. Studies were considered homogenous if *P* > 0.05 and I^2^ ≤ 50%, and we performed only pooled sensitivity, specificity, positive likelihood ratio (PLR), negative likelihood ratio (NLR), and diagnostic odds ratio (DOR). If *P* < 0.05 or I^2^ ≥ 50%, indicating statistically significant heterogeneity between the studies, further meta-regression and subgroup analysis were carried out to determine the possible sources of heterogeneity.

Sensitivity, specificity, accuracy, positive and negative predictive values, PLR, NLR, and DOR of VOCs in the diagnosis of digestive system neoplasm were obtained from the individual study, and forest plots were used to calculate and graphically display pooling of the data. The SROC curve was displayed to obtain the optimal diagnostic efficiency for VOCs, and the area under the summary ROC curve (AUC) was calculated. The publication bias was presented by using the Deeks’ funnel plot asymmetry test. Meta-analysis was performed using STATA15.0 (StataCorp, College Station, TX, USA) with the MIDAS module, while RevMan 5.4 (Revman, the Cochrane Collaboration) was used to evaluate the quality of the included studies. A *P*-value of < 0.05 was considered statistically significant.

## Results

### Literature search

**Figure 1:**
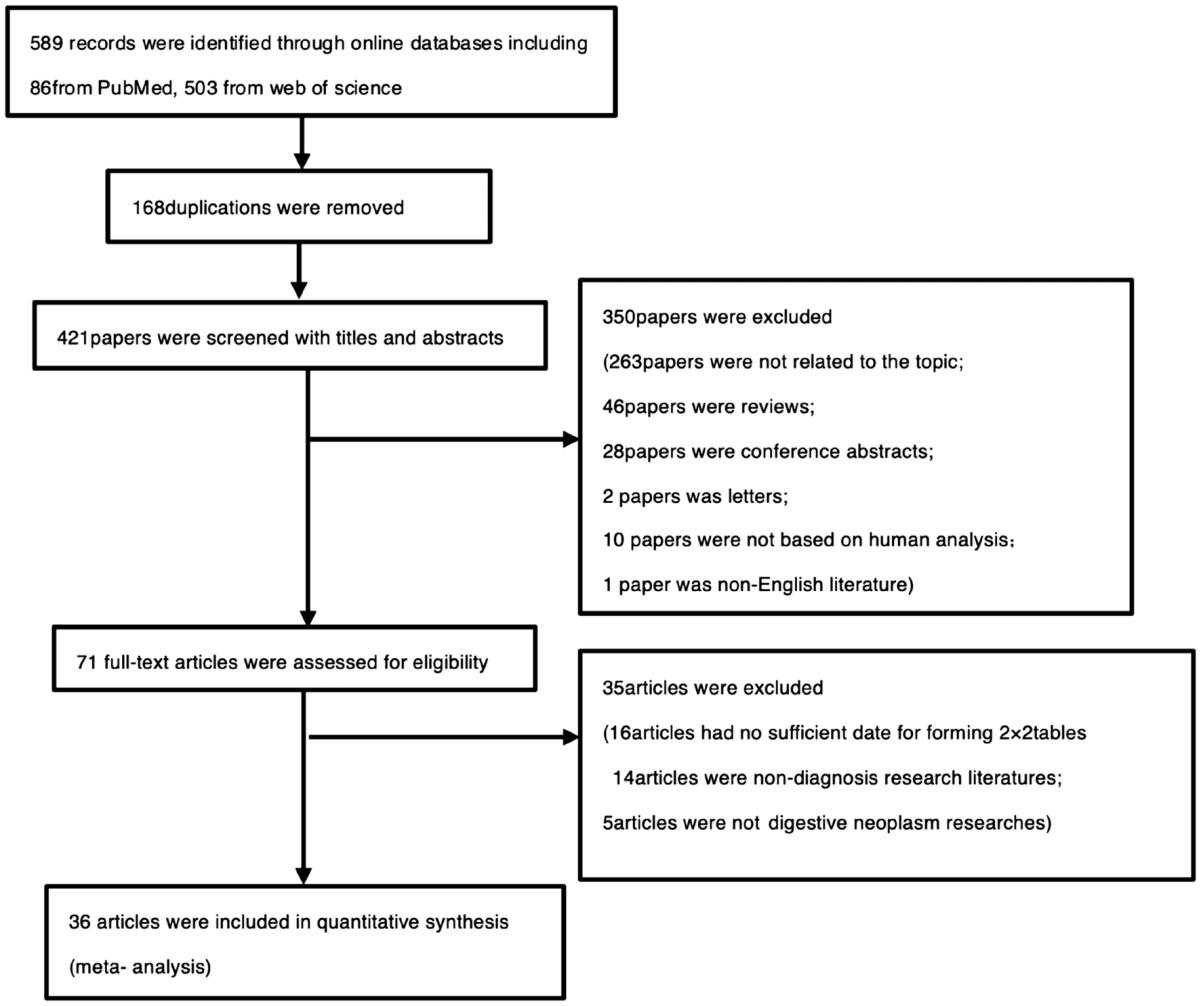
Flow diagram of the study selection process.

The initial search of PubMed and Web of Science databases yielded 589 articles. Of these, 168 were excluded due to repetitive publications, and 350 articles were excluded based on the inclusion/exclusion criteria after reading the titles and abstracts: 263 did not associate VOCs as tools for the diagnosis of neoplasm of the digestive system; 46 were review articles; 28 were conference abstracts; 2 were letters; 10 were not based on human studies; 1 was non-English literature. A total of 35 articles were excluded after reading the full text: 14 were not of diagnostic research; 5 were research of non-digestive tract neoplasm; 16 articles contained insufficient data to form 2 × 2 tables. Finally, a total of 36 studies were included in our meta-analysis (Fig. 1).

### Study characteristics

The 36^12,29–63^ included studies comprised of a total of 39 datasets that investigated 5 cancer types: 3 on liver cancer, 16 on colorectal cancer, 10 on gastric cancer, 4 on esophageal cancer, and 6 on pancreatic cancer. Overall, our meta-analysis was performed based on 1712 cases and 3215 controls from 11 different countries. As for the origin of the VOCs measurements, 4 datasets measured VOCs patterns in feces, 22 (from 21 studies) in exhaled breaths, 8 (from 7 studies) in urine, 1 in blood, 2 (from 1 study) in saliva, 1 in bowel gas, and 1 in bile.

There were 2 datasets that used the electronic nose as the analytical platform, 16 datasets (from 15 studies) used gas chromatography coupled with mass spectrometry (GC–MS), 5 datasets used field asymmetric ion mobility spectrometer (FAIMS), 8 datasets (from 7 studies) used selected ion flow tube mass spectrometer (SIFT-MS), and 8 studies used other analytical platforms: 2 datasets used silicon nanowire field-effect transistor (SINW-FET), 1 dataset used proton transfer reaction mass spectrometer (PTR-MS), 1 dataset used ion–molecule reaction mass spectrometry (IMR-MS), 1 dataset used single-photon ionization mass spectrometry (SPI-MS), 1 dataset used field asymmetric ion mobility spectrometer–mass spectrometry (FAIMS–MS) and 2 datasets used sensor (Table 1).

**Table 1:** Major characteristics of included studies.

Author	Year	Country	Gender (%male)	Mean age	Tp	Fp	Fn	Tn	Cancer type	VOC sources	Analytical platform	Control type	Sample size
Xue et al.^27^	2008	China	100	50.5	18	0	1	18	Liver cancer	Blood	GC–MS	Health	37
Qin et al.^28^	2010	China	76	50.9	26	3	4	33	Liver cancer	Exhaled breath	GC–MS	Health	66
Altomare et al.^29^	2013	Italy	42	55	32	7	5	34	Colorectal cancer	Exhaled breath	GC–MS	Health	78
Xu et al.^30^	2013	China	38	53.5	33	9	4	84	Gastric cancer	Exhaled breath	GC–MS	Non-cancer	130
Arasaradnam et al.^31^	2014	U.K	56	53.5	73	20	10	30	Colorectal Cancer	Urine	FAIMS	Health	133
de Meij et al.^32^	2014	Netherlands	32	60	34	7	6	50	Colorectal cancer	Feces	GC–MS	Health	97
Batty et al.^33^	2015	U.K	–	–	24	9	7	22	Colorectal cancer	Feces	SIFT-MS	Health	62
Bhatt et al.^34^	2015	America	54	59.4	17	2	3	17	Esophageal Adenocarcinoma	Exhaled breath	SIFT-MS	Non-cancer	39
Kumar et al. **I**^12^	2015	U.K	63	61.2	33	13	0	100	Gastric cancer	Exhaled breath	SIFT-MS	Non-cancer	146
Kumar et al.**II**^12^	2015	U.K	67	64	47	22	1	107	Esophageal Adenocarcinoma	Exhaled breath	SIFT-MS	Non-cancer	177
Shehada et al.^35^	2015	Latvia	–	–	5	2	2	17	Gastric cancer	Exhaled breath	SINW-FET	Non-cancer	26
Amal et al.^36^	2016	Latvia	–	63	17	2	3	34	Colorectal cancer	Exhaled breath	GC–MS	Health	56
Chen et al.^37^	2016	China	72	45	121	3	23	53	Gastric cancer	Exhaled breath	GC–MS	Health	200
Shehada et al.^38^	2016	Latvia; U.K; Israel	79	62.5	35	3	5	126	Gastric cancer	Exhaled breath	SINW-FET	Health	169
Zou et al.^39^	2016	China	47	58.4	25	6	4	51	Esophageal cancer	Exhaled breath	PTR-MS	Health	86
Arasaradnam et al.^40^	2018	UK	42	57.9	74	14	7	67	Pancreatic cancer	Urine	FAIMS	Health	162
Duran-Acevedo et al.^41^	2018	Colombia	59	69.8	14	1	0	14	Gastric cancer	Exhaled breath	GC–MS	Non-cancer	29
Ishibe et al.^42^	2018	Japan	70	50	27	11	3	15	Colorectal cancer	Bowel gas	GC–MS	Health	56
Markar et al.^43^	2018	U.K	61	63	26	13	6	19	Pancreatic cancer	Exhaled breath	GC–MS	Non-cancer	64
Markar et al.^44^	2018	U.K	64	–	130	33	33	139	Esophagogastric cancer	Exhaled breath	SIFT-MS	Health	335
Princivalle et al.^45^	2018	Italy	52	57	65	16	0	86	pancreatic cancer	Exhaled breath	IMR-MS	Health	167
Schuermans et al.^46^	2018	China	50	47	13	8	3	20	Gastric cancer	Exhaled breath	E-nose	Health	44
Widlak et al.^47^	2018	U.K	–	–	22	86	13	147	Colorectal cancer	Urine	FAIMS	Health	268
Bond et al.^48^	2019	U.K	40	67.3	18	9	3	51	Colorectal cancer	Feces	GC–MS	Health	81
Broza et al.^49^	2019	Latvia	–	–	3	153	0	570	Gastric cancer	Exhaled breath	Sensor	Non-cancer	726
Markar et al.^50^	2019	U.K	–	–	21	8	4	46	Colorectal cancer	Exhaled breath	SIFT-MS	Non-cancer	79
McFarlane et al.^51^	2019	U.K	47	58.7	39	25	17	57	Colorectal cancer	Urine	FAIMS-MS	Health	138
Mozdiak et al. **I**^52^	2019	U.K	–	–	8	4	2	20	Colorectal cancer	Urine	GC–MS	Health	34
Mozdiak et al. **II**^52^	2019	U.K	–	–	12	1	0	11	Colorectal cancer	Urine	FAIMS	Health	24
Nissinen et al.^53^	2019	Finland	50	64.5	54	11	14	41	Pancreatic Cancer	Urine	FAIMS	Health	120
Altomare et al.^54^	2020	Italy	–	–	74	6	8	81	Colorectal cancer	Exhaled breath	GC–MS	Health	169
Bel'skaya et al. **I**^55^	2020	Russia	–	–	9	0	2	16	Gastric cancer	Saliva	GC–MS	Health	27
Bel'skaya et al. **II**^55^	2020	Russia	–	–	17	0	1	16	Colorectal cancer	Saliva	GC–MS	Health	34
Hong et al.^56^	2020	China	–	–	28	1	1	23	Gastric cancer	Exhaled breath	SPI-MS	Health	53
Miller-Atkins et al.^57^	2020	America	–	–	67	46	25	114	Liver cancer	Exhaled breath	SIFT-MS	Non-cancer	252
Navaneethan et al.^58^	2020	America	58	62.9	19	0	0	12	Pancreatic Cancer	Bile	SIFT-MS	Non-cancer	31
van Keulen et al.^9^	2020	Netherlands	61	–	16	4	13	23	Colorectal cancer	Exhaled breath	E-nose	Health	56
Zonta et al.^60^	2020	Italy	–	–	116	46	22	214	Colorectal cancer	Feces	Sensor	Health	398
Daulton et al.^61^	2021	U.K	47	57	38	2	7	31	Pancreatic cancer	Urine	GC–MS	Health	78

*VOCs* volatile organic compounds, *GC–MS* gas chromatography and mass spectrometry, *FAIMS* field asymmetric ion mobility spectrometer, *SIFT-MS* selected ion flow tube mass spectrometer, *SINW-FET* silicon nanowire field effect transistor, *PTR-MS* proton transfer reaction mass spectrometer, *IMR-MS* ion–molecule reaction mass spectrometry, *SPI-MS* single photon ionization mass spectrometry, *Tn* true negative, *Tp* true positive, *Fp* false positive, *Fn* false negative.

### Risk of bias and quality assessment

There was no apparent publication bias between the included studies, given that the funnel chart was well-proportioned with a *P*-value of greater than 0.39 (see Supplemental Fig. S1). The analysis using the QUADAS-2 tool showed a low risk of bias and moderate to a high quality of the included studies (see Supplemental Fig. S2).

### Diagnostic effect

**Figure 2:**
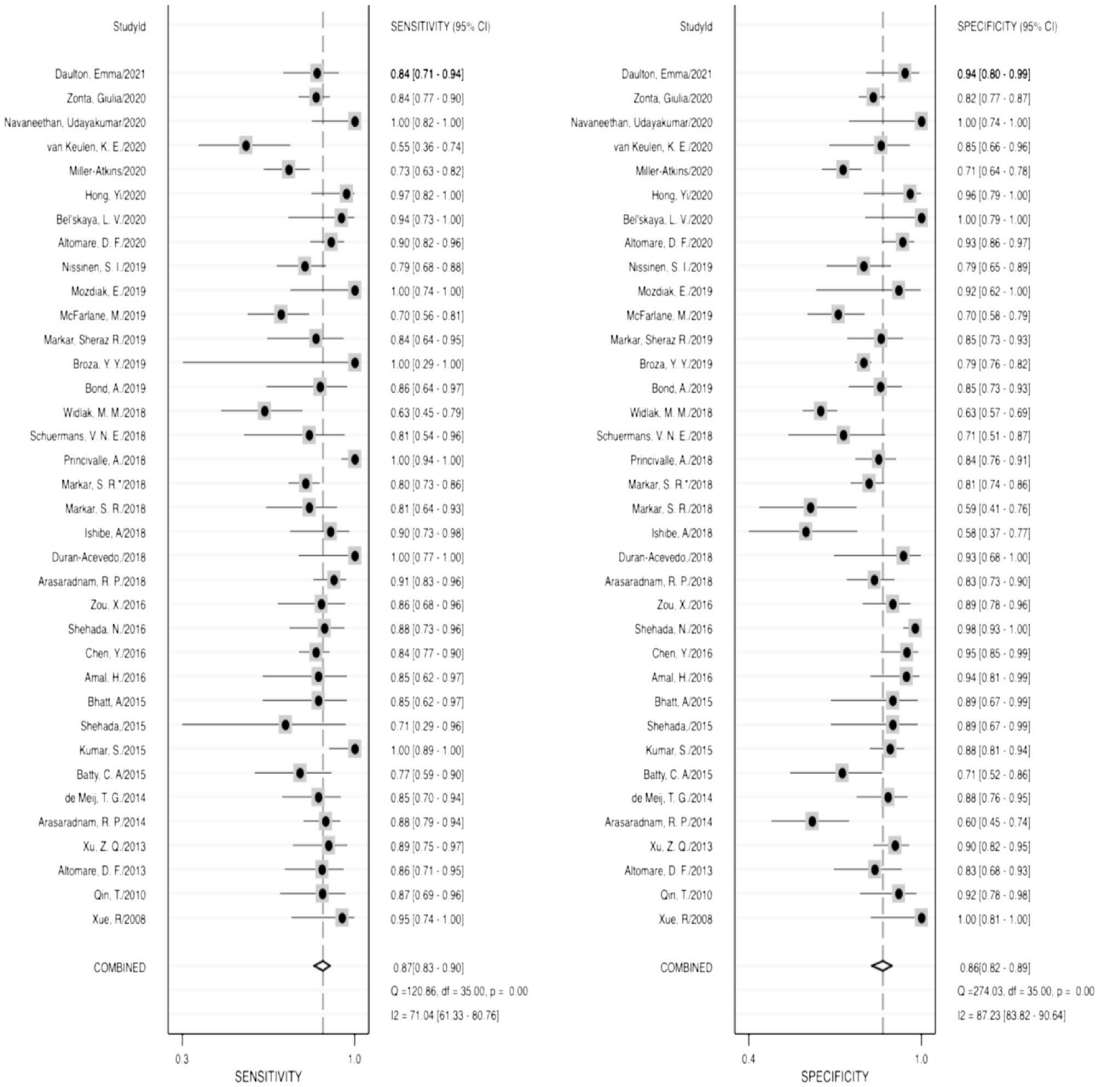
Forest plots of pooled sensitivity and specificity.

**Figure 3:**
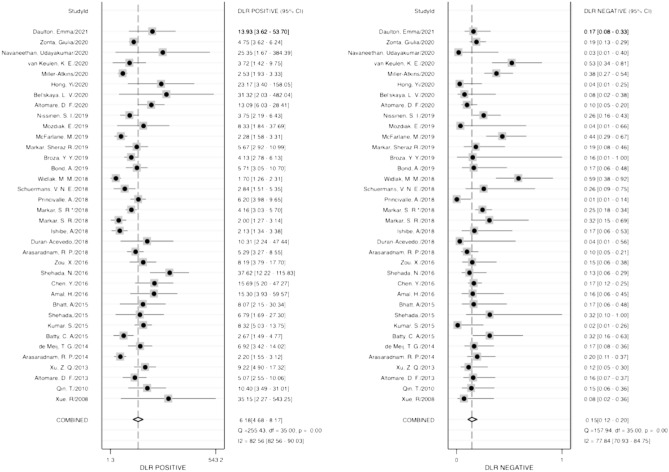
Forest plots of pooled positive likelihood radio and negative likelihood ratio.

**Figure 4:**
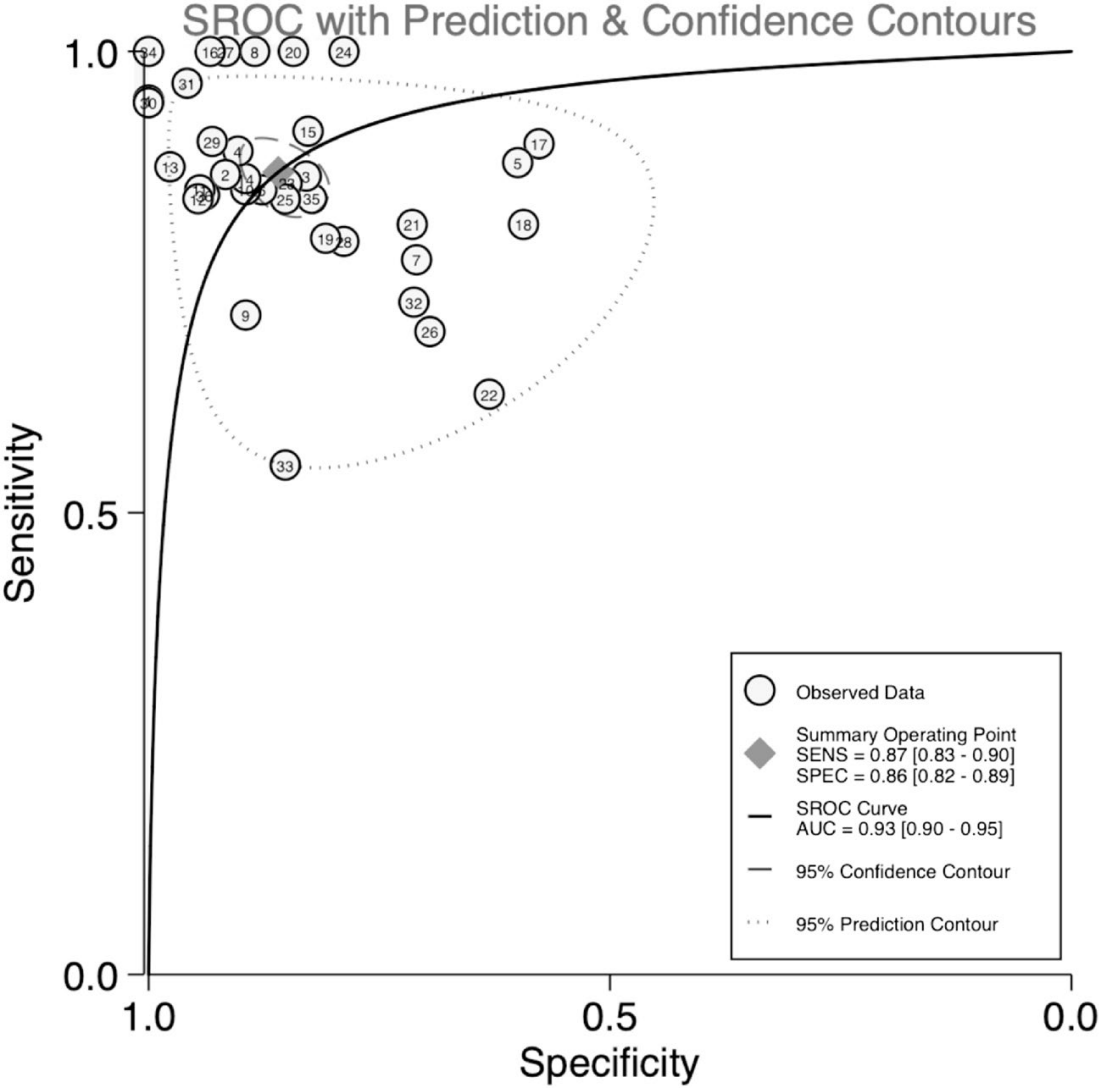
SROC curve of VOCs for the diagnosis of digestive system cancer. Abbreviations: The numbers in the circles represent the studies included in the paper. The eighth study corresponds to reference^12^, and the remaining studies^1–11,13–36^ correspond to reference^27–61^. VOCs: Volatile organic compounds.

Diagnostic accuracy was estimated using several indicators, including sensitivity, specificity, PLR, NLR, and DOR. The pooled sensitivity and specificity of VOCs for diagnosing neoplasm of the digestive system were 0.87 (95% CI 0.83–0.90) and 0.86 (95% CI 0.82–0.89), respectively (Fig. 2), while the pooled PLR and NLR were 6.18 (95% CI 4.68–8.17) and 0.15 (95% CI 0.12–0.20), respectively (Fig. 3). Furthermore, the pooled DOR (see Supplemental Fig. S3) and AUC (Fig. 4) were 40.61 (95% CI 24.77–66.57) and 0.93 (95% CI 0.90–0.95), respectively.

### Heterogeneity

Heterogeneity existed in the pooled specificity (I^2^ = 71.04%, *P* = 0.00), as well as the pooled results of sensitivity (I^2^ = 87.23%, *P* = 0.00) (Fig. 2).

### Meta-regression and subgroup analysis

Univariate meta-regression and subgroup analyses were performed to investigate potential sources of heterogeneity between studies. The forest plots (see Supplemental Fig. S4) of univariate meta-regression indicated that race, sample size, analytical platforms (MS, ISM, Sensor), VOCs source (exhaled breath, feces, urine, others), and the control groups (healthy individuals and non-cancer/benign disease cohort) could be the sources of the heterogeneity. Factors including race, analytical platform, and VOCs source were included in the subgroup analysis (Table 2). Both the sensitivity and specificity in Asian (0.88, 95% CI 0.82–0.92; and 0.91, 95% CI 0.81–0.96, respectively) were higher than that in European and American (0.86, 95% CI 0.82–0.90; and 0.84, 95% CI 0.80–0.88, respectively). With regards to the source of VOCs, the sensitivity and specificity (0.87, 95% CI 0.82–0.91; and 0.87, 95% CI 0.83–0.91, respectively) were the highest when VOCs were derived from exhaled breaths in the screening for tumors of the digestive system tumors. For the analytical platform, the sensitivity and specificity (0.89, 95% CI 0.85–0.92; and 0.88, 95% CI 0.84–0.91, respectively) were the highest when VOCs were analyzed with MS.

**Table 2:** Subgroup analysis of diagnostic effect.

Subgroup	No. studies	No. sample sizes	Sensitivity value	Specificity value
**Race**				
European and American	27	3983	0.86 (0.82–0.90)	0.84 (0.80–0.88)
Asian	9	706	0.88 (0.82–0.92)	0.91 (0.81–0.96)
**VOCs source**				
Exhaled breath	21	3001	0.87 (0.82–0.91)	0.87 (0.83–0.91)
Faeces	4	638	0.83 (0.78–0.88)	0.83 (0.79–0.86)
Urine	7	933	0.82 (0.73–0.88)	0.76 (0.67–0.83)
Other source	4	158	–	–
**Analysis platform**				
MS	25	2459	0.89 (0.85–0.91)	0.88 (0.84–0.91)
IMS	5	707	0.86 (0.73–0.93)	0.75 (0.64–0.84)
Sonsers	6	1419	0.79 (0.67–0.88)	0.86 (0.75–0.93)

Data selection in subgroup analysis of race group: Samples in three studies were from the same race group. My data extraction: Kumar S 2015 selected gastric cancer samples; Mozdiak E 2019 selects FAIMS samples; Bel'skaya LV 2020 selected colorectal cancer sample.

Data selection in subgroup analysis of VOCS source: Samples in three studies were from the same VOCs source group. My data extraction: Kumar S 2015 selected esophageal Adenocarcinoma samples; Mozdiak E 2019 selected GC–MS samples; Bel'skaya LV 2020 selected colorectal cancer sample.

Data selection in subgroup analysis of analysis platform: My data extraction: Data from two different analysis platforms in Kumar S 2015 and Mozdiak E 2019 were extracted; The platform of McFarlane M 2019 was combined with FAIMS and MS, so it was not included in any group of the analysis platform subgroup; Because two sets of data in the study of Bel'skaya LV were based on GC–MS platform, we selected colorectal cancer samples.

*MS* mass spectrometry, *IMS* ion mobility spectrometer, *VOCs* volatile organic compounds.

## Discussion

VOCs as biomarkers have been explored in recent years and are regarded as a new frontier in cancer diagnosis. It has great potentials in developing into a rapid, noninvasive, and inexpensive cancer diagnostic tool^64,65^. A recent meta-analysis has demonstrated a sensitivity of 0.79 and specificity of 0.89 in diagnosing cancer using VOCs analysis^24^. Also, in diagnosing colorectal cancer, another meta-analysis has revealed the sensitivity and specificity of VOCs being 0.82 and 0.79, respectively^26^. Compared with the study by Zhou et al.^26^ that included studies of a single tumor, our meta-analysis included clinical studies of five different tumor types of the digestive system. Also, contrary to the study by Hanna et al.^24^, the sources of VOCs in our study were not limited to exhaled gases only but also included feces, blood, and urine. Furthermore, the number of studies included in our meta-analysis was also higher.

From the retrieved data of the included studies, we performed calculations on the sensitivity, specificity, PLR, NLR, and DOR to estimate the diagnostic accuracy of VOCs as a screening tool for tumors of the digestive system. From our analysis, the pooled sensitivity and specificity were 0.87 and 0.86, respectively. Moreover, the overall diagnostic performance was assessed using the SROC curve, which revealed an AUC of 0.93, indicating excellent diagnostic performance. The DOR represented a single measure of test accuracy, which in our analysis, the DOR was 40.61 (DOR > 10), indicating great discriminatory test capability. Furthermore, the likelihood ratio and post-test probability indicated the risk of tumors of the digestive system when tested positive or negative. Our analysis revealed a PLR of 6, indicating that patients with digestive system tumors were six times more likely to test positive than healthy individuals. Coupled with the NLR of 0.15, VOCs analysis represented a promising method to diagnose the neoplasm of the digestive system.

To identify potential sources of heterogeneity, univariate meta-regression and meta-analysis were performed, which revealed that factors including race, sample size, source of VOCs, analytical platforms, and types of the control group might be sources of heterogeneity in our meta-analysis. Further analysis demonstrated higher specificity for VOCs in the screening of tumors in the digestive system tumors of the Asians than the others (Europeans and Americans), suggesting that the performance of VOCs in cancer screening may vary according to race or ethnicity. Also, we found that analysis of VOCs from the exhaled breath performed better than other sources of VOCs. However, the sample size of studies investigating other sources of VOCs was smaller compared with those of VOCs from exhaled breath. For the analytical platform, the use of MS (mass spectrometry) to analyze VOCs had the highest sensitivity and specificity compared with other platforms. However, the sample size of studies utilizing MS was also much larger than that of other analytical platforms. In our study, the performance of VOCs as a screening tool for different tumors was not analyzed, given that our objective was to explore VOCs analysis as a rapid and non-invasive screening tool for tumors of the digestive system, with its applicability as a primary population screening rather than as a diagnostic tool for individual tumor types.

There were several reasons why this meta-analysis did not conduct threshold effect analysis: (1) different studies used different instruments for analyzing organic matter; (2) different instruments detected different types of organic matter; (3) different studies had different ways of setting the threshold value of organic compounds. Therefore, it was difficult to analyze the diagnostic ability of specific organic compounds for tumors. However, this did not affect the results that VOCs have good diagnostic ability in the digestive system.

Compared with other tumor markers currently applied in clinical practice, VOCs have demonstrated superior diagnostic ability in screening for gastric cancer and colorectal cancer. Serum pepsinogen is a widely used biomarker with good diagnostic efficacy for gastric cancer screening, with a meta-analysis demonstrating a sensitivity of 0.69 and a specificity of 0.73, while the sensitivity and specificity could be improved further to 0.70 and 0.79 respectively when applying the ratio of PG I to PG II concentrations^66^. Besides, the combination of CA199 with CA242 has demonstrated a sensitivity of 0.90 and a specificity of 0.76^67^. From our analyses, the sensitivity (0.90) and specificity (0.91) of VOCs for gastric cancer, and the sensitivity (0.84) and specificity (0.82) of VOCs for colorectal cancer exhibited superior performance to conventional biomarkers in screening for neoplasm.

There were limitations to our study. The included studies were mostly of case–control and cross-sectional nature, with few prospective longitudinal studies to associate VOCs with disease severity. Second, only articles in the English language had been selected, which invariably resulted in selection bias. Third, the cancer types, and analytical platforms of tumor-associated VOCs also varied between the studies. Therefore, we did not analyze the performance of varying VOCs in screening for different tumors of the digestive system.

Therefore, further studies are warranted to not only innovate and combine advanced technologies in the sampling, detection, and analysis of VOCs but also to standardize these methods. Moreover, it is vital to explore the differences of VOCs between varying types and stages of tumors. Clinical trials would then be required for the validation of results before widespread clinical application.

## Conclusion

The pooled results of our meta-analysis have confirmed the difference in the VOCs analysis between patients with tumors of the digestive system and healthy individuals, which sheds light on VOCs analysis as a promising novel screening tool for early detection of tumors.

## Supplementary Information

Supplementary Information 1.

Supplementary Figure S1.

Supplementary Figure S2.

Supplementary Figure S3.

Supplementary Figure S4.

## Data Availability

All data generated or analyzed during this study are included in this published article and its supplementary information.
