# Supplementary material for: Volatile organic compounds as a potential screening tool for neoplasm of the digestive system: a meta-analysis
Source: Sci Rep. 2021 Dec 9;11:23716. doi: 10.1038/s41598-021-02906-8 (PMC8660806; doi:10.1038/s41598-021-02906-8)
Supplement: Supplementary file 1 — Supplementary Information 1. [file 41598_2021_2906_MOESM1_ESM.pdf]

Volatile organic compounds as a potential screening tool for neoplasm of the digestive system: A meta-analysis

Lixing Wang, MD[1], Junan Li, MD[2], Xiaoliang Xiong, MD[1], Tingting Hao, MD[1], Chao Zhang, MD[1], Zhao Gao, MD[1], Lili Zhong, PhD[3#], Yinlong Zhao, PhD[1#]

1. Department of Nuclear Medicine, The Second Hospital of Jilin University, Changchun 130041, China
2. Gastroenterology and Center of Digestive Endoscopy, The Second Hospital of Jilin University, Changchun 130041, China
3. Jilin Provincial Key Laboratory on Molecular and Chemical Genetic, The Second Hospital of Jilin University, Changchun 130041, China

# The authors contributed equally to this work, and they are both the corresponding author.

Corresponding E-mail: yinlong@jlu.edu.cn

STATA Operation Details

We used midas in STATA software to summarize data and output graphics.

1.Enter raw data (including author, year, tp, fp, tn, fn) in the Data Editor

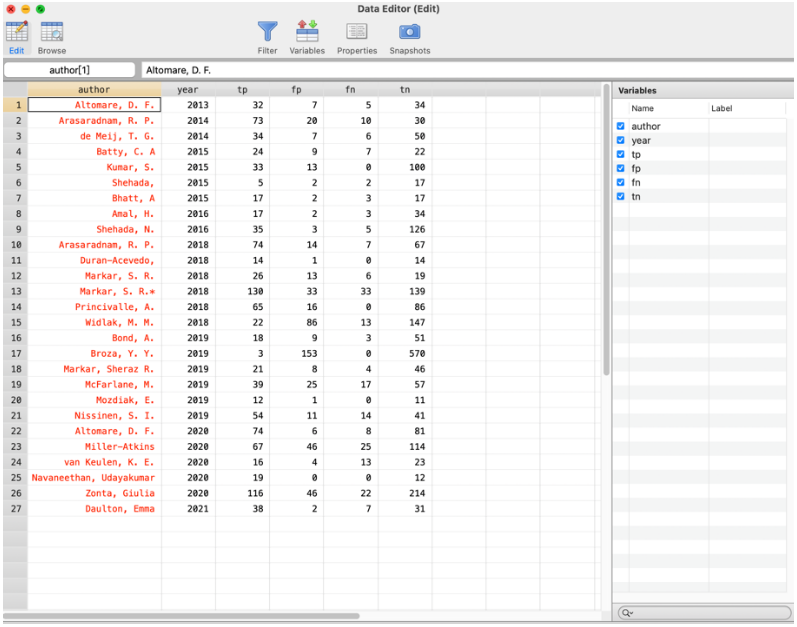

	author	year	tp	fp	fn	tn
1	Altomare, D. F.	2013	32	7	5	34
2	Arasaradnam, R. P.	2014	73	20	10	30
3	de Meij, T. G.	2014	34	7	6	50
4	Batty, C. A	2015	24	9	7	22
5	Kumar, S.	2015	33	13	0	100
6	Shehada,	2015	5	2	2	17
7	Bhatt, A	2015	17	2	3	17
8	Amal, H.	2016	17	2	3	34
9	Shehada, N.	2016	35	3	5	126
10	Arasaradnam, R. P.	2018	74	14	7	67
11	Duran-Acevedo,	2018	14	1	0	14
12	Markar, S. R.	2018	26	13	6	19
13	Markar, S. R.*	2018	130	33	33	139
14	Princivalle, A.	2018	65	16	0	86
15	Widlak, M. M.	2018	22	86	13	147
16	Bond, A.	2019	18	9	3	51
17	Broza, Y. Y.	2019	3	153	0	570
18	Markar, Sheraz R.	2019	21	8	4	46
19	McFarlane, M.	2019	39	25	17	57
20	Mozdiak, E.	2019	12	1	0	11
21	Nissinen, S. I.	2019	54	11	14	41
22	Altomare, D. F.	2020	74	6	8	81
23	Miller-Atkins	2020	67	46	25	114
24	van Keulen, K. E.	2020	16	4	13	23
25	Navaneethan, Udayakumar	2020	19	0	0	12
26	Zonta, Giulia	2020	116	46	22	214
27	Daulton, Emma	2021	38	2	7	31

2. Enter the code in the command.

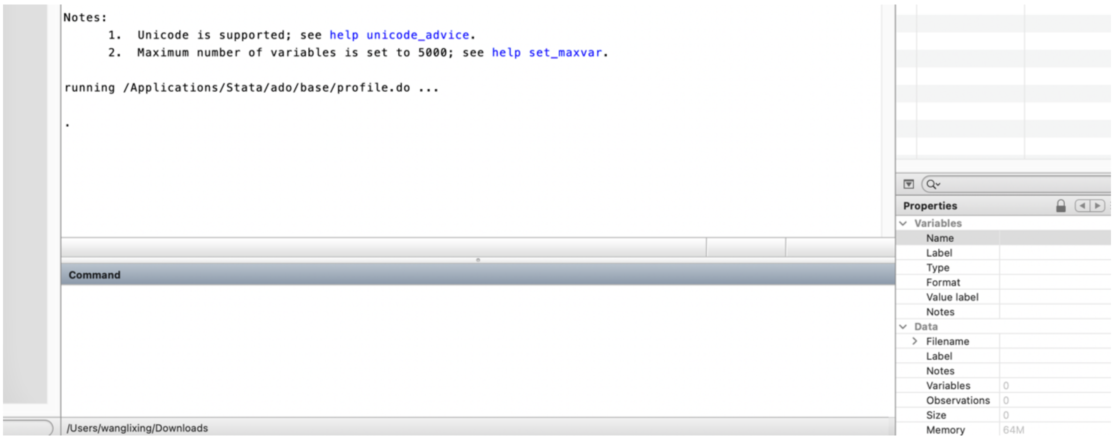

3.Input “midas tp fp fn tn, id(author year) ms(0.75) ford fors bfor(dss)”, and get a forest plot of pooled sensitivity and specificity.

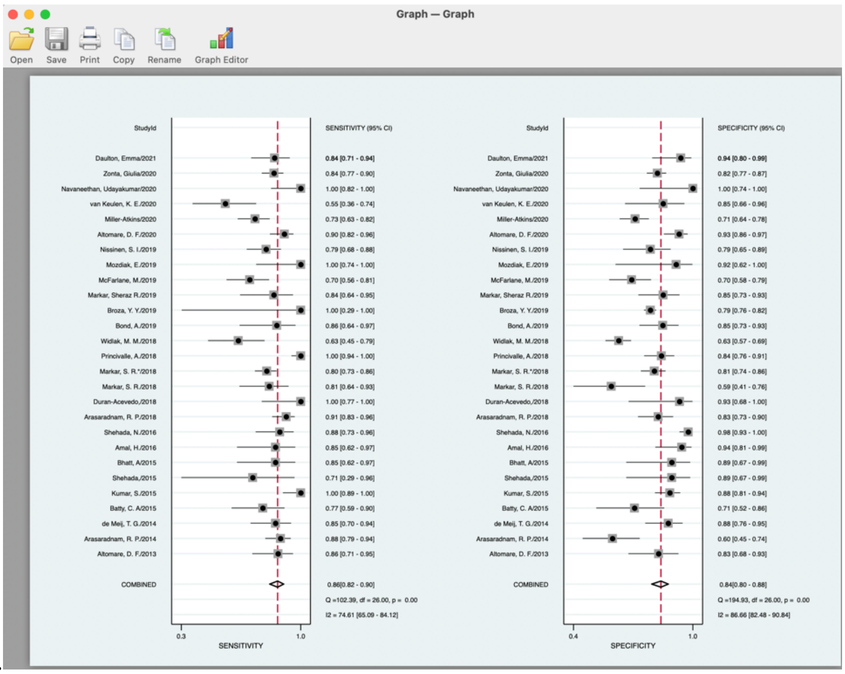

.

4. Input “midas tp fp fn tn, id(author year) ms(0.75) ford fors bfor(dss)”,and get the Summary ROC Curve.

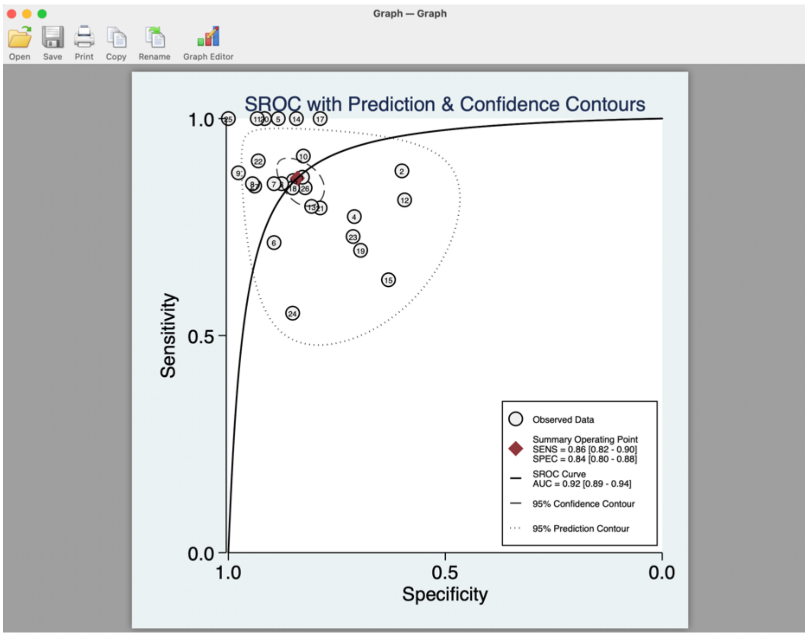

6. Input “midas tp fp fn tn, id(author year) ms(0.75) ford fors bfor(dlr)”, and get a forest plot of the pooled PLR and NLR.

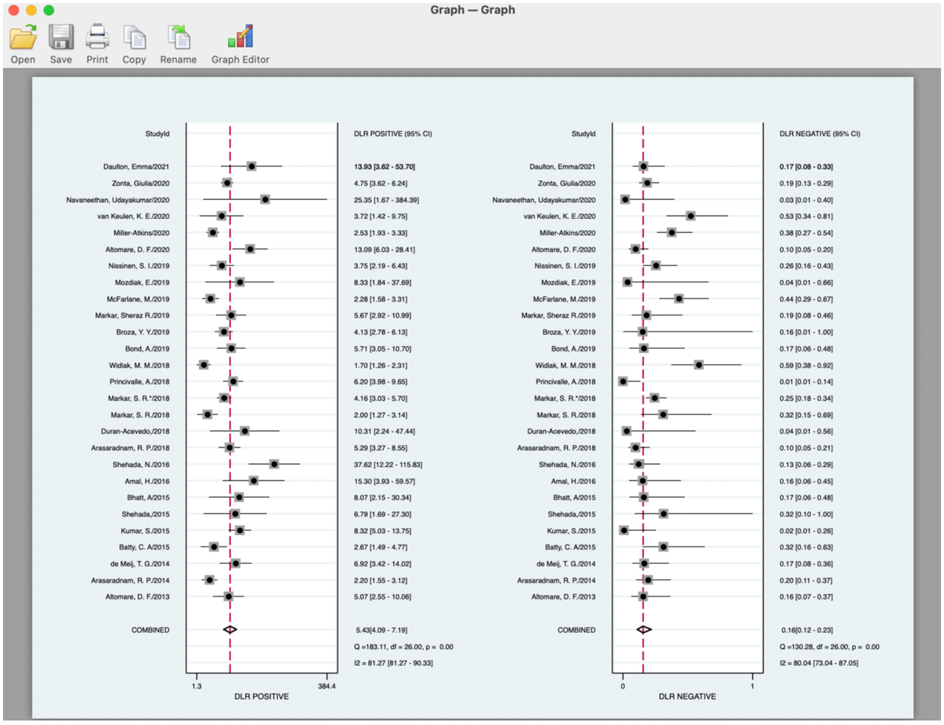

7. Input “midas tp fp fn tn, id(author year) ms(0.75) ford fors bfor(dlor)”, and get a forest plot of the pooled the pooled DOR.

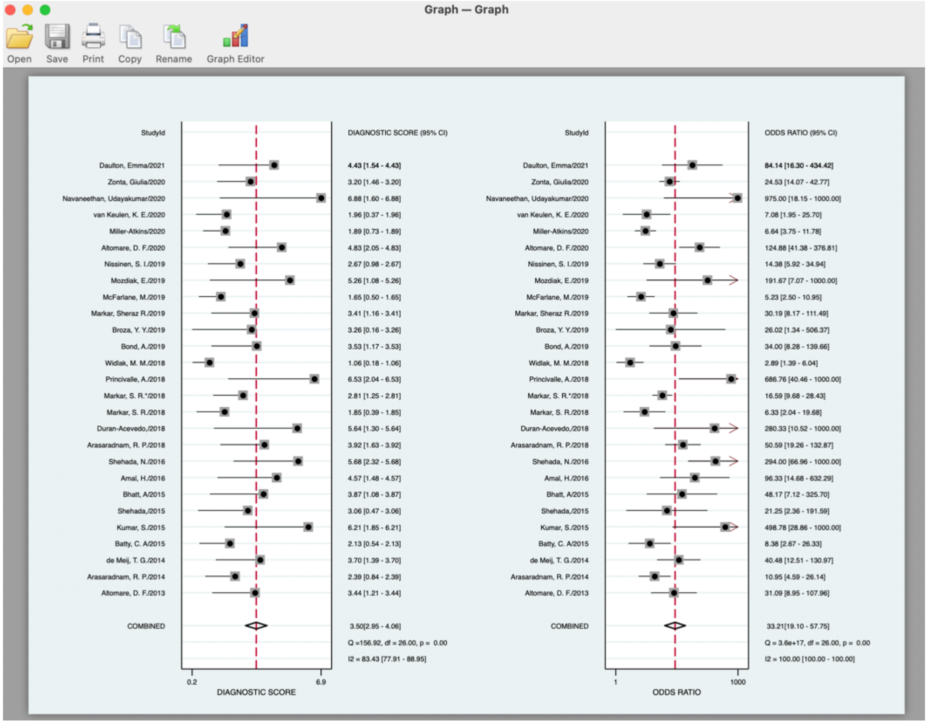

8. Input “midas tp fp fn tn, pubbias”,and get a Deeks’Funnel Plot.

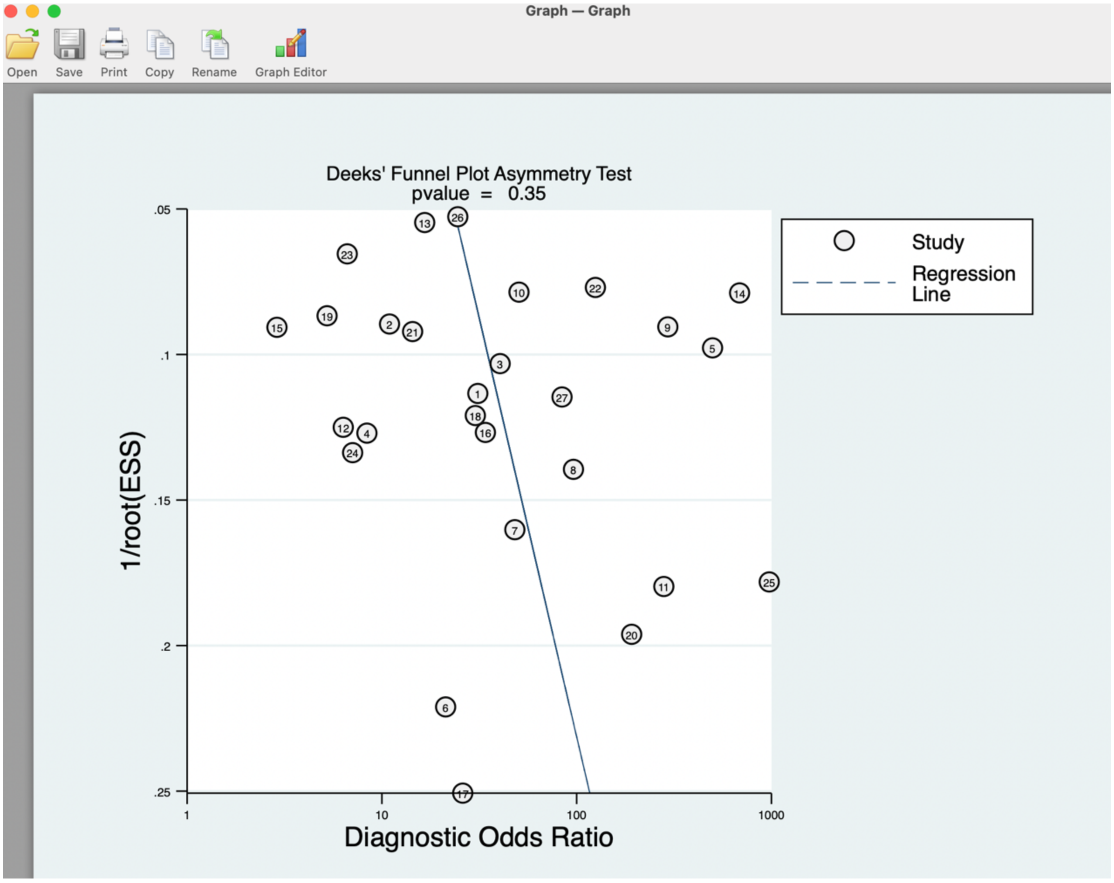

9. After assigning dichotomous variables to the data for meta-regression,Input “midas tp fp fn tn, reg(prodesign ssize30 fulverif testdescr refdescr subjdescr report brdspect blinded”,and get the forest plots of univariate meta-regression.

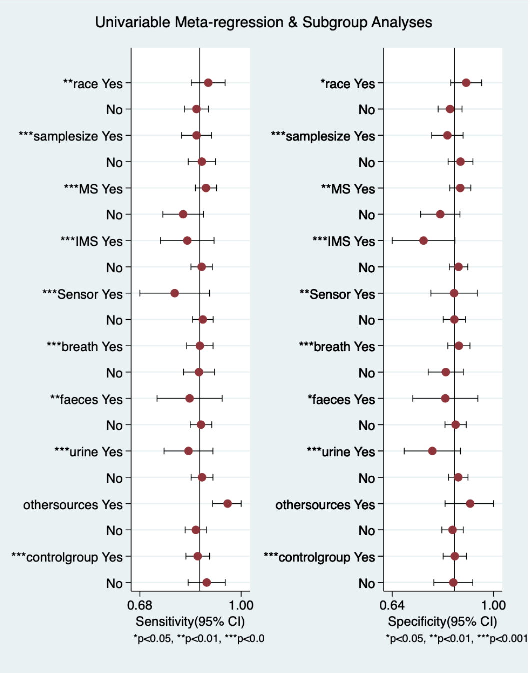
Univariable Meta-regression & Subgroup Analyses
**race Yes
No
***samplesize Yes
No
***MS Yes
No
***IMS Yes
No
***Sensor Yes
No
***breath Yes
No
**faeces Yes
No
***urine Yes
No
othersources Yes
No
***controlgroup Yes
No
0.68
1.00
Sensitivity(95% CI)
*p<0.05, **p<0.01, ***p<0.0
*race Yes
No
***samplesize Yes
No
**MS Yes
No
***IMS Yes
No
**Sensor Yes
No
***breath Yes
No
*faeces Yes
No
***urine Yes
No
othersources Yes
No
***controlgroup Yes
No
0.64
1.00
Specificity(95% CI)
*p<0.05, **p<0.01, ***p<0.001
