# Supplementary material for: Volatile organic compounds as a potential screening tool for neoplasm of the digestive system: a meta-analysis
Source: Sci Rep. 2021 Dec 9;11:23716. doi: 10.1038/s41598-021-02906-8 (PMC8660806; doi:10.1038/s41598-021-02906-8)
Supplement: Supplementary file 2 — Supplementary Figure S1. [file 41598_2021_2906_MOESM2_ESM.docx]

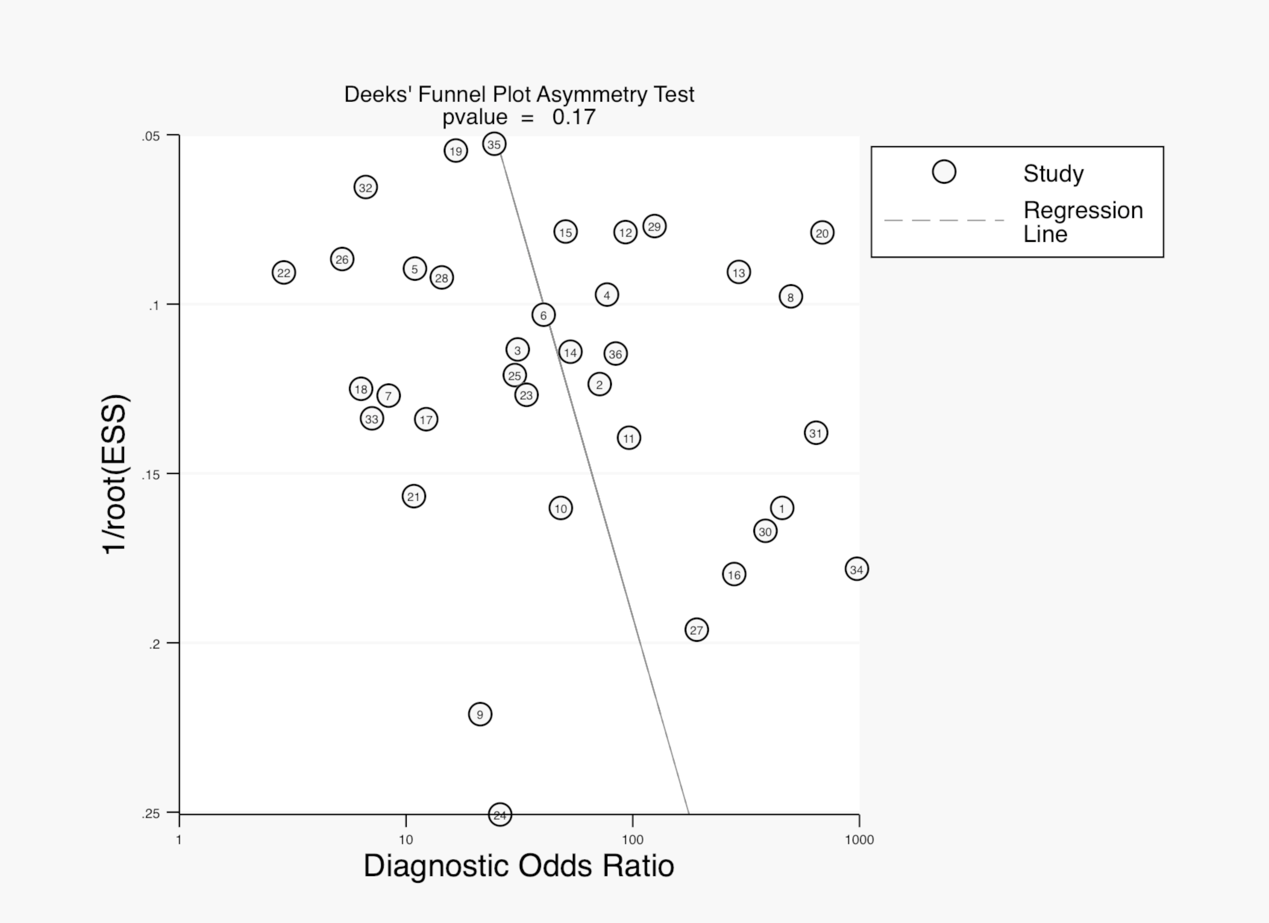


Supplemental figure 1. Deek funnel plot for the assessment of the publication bias.

Abbreviations: The numbers in the circles represent the studies included in the paper. The eighth study corresponds to reference 12, and the remaining studies (1-11, 13-36) correspond to reference 27-61.
