# Supplementary material for: Volatile organic compounds as a potential screening tool for neoplasm of the digestive system: a meta-analysis
Source: Sci Rep. 2021 Dec 9;11:23716. doi: 10.1038/s41598-021-02906-8 (PMC8660806; doi:10.1038/s41598-021-02906-8)
Supplement: Supplementary file 3 — Supplementary Figure S2. [file 41598_2021_2906_MOESM3_ESM.docx]

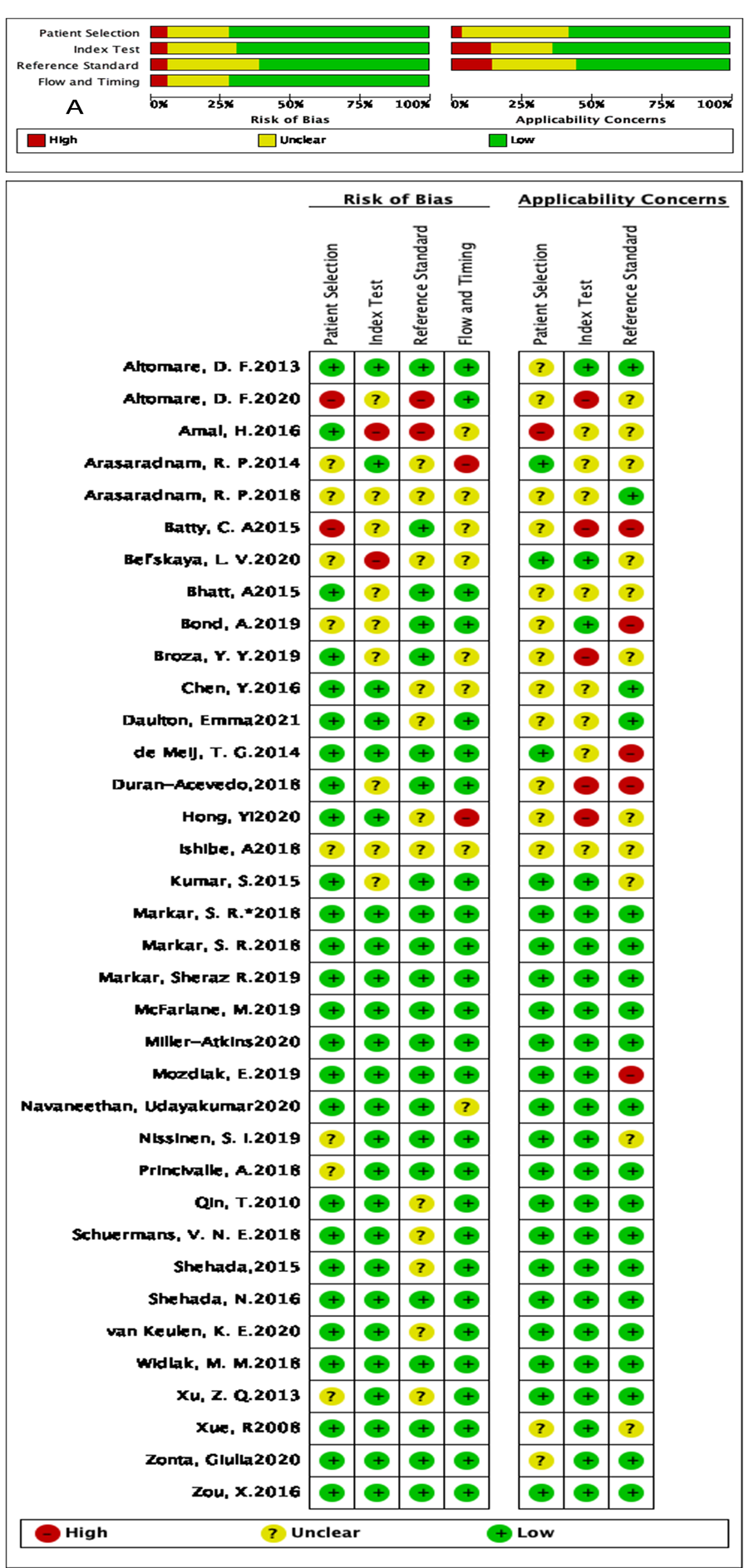


Supplemental figure 2. A risk of bias and applicability concerns graph, B risk of bias and applicability concerns summary
