# Supplementary material for: Volatile organic compounds as a potential screening tool for neoplasm of the digestive system: a meta-analysis
Source: Sci Rep. 2021 Dec 9;11:23716. doi: 10.1038/s41598-021-02906-8 (PMC8660806; doi:10.1038/s41598-021-02906-8)
Supplement: Supplementary file 4 — Supplementary Figure S3. [file 41598_2021_2906_MOESM4_ESM.docx]

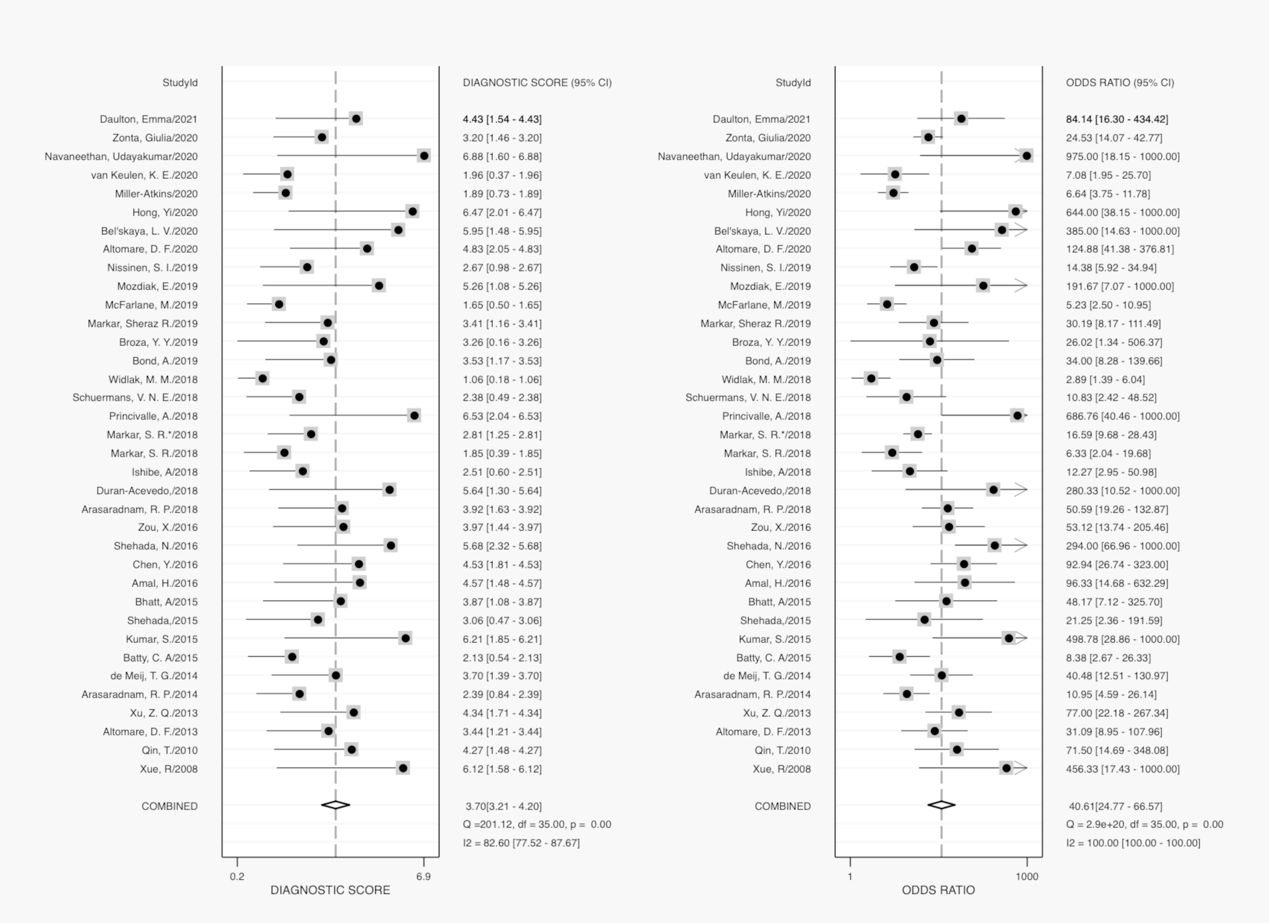


Supplemental figure 3. Forest plots of DOR of VOCs for the diagnosis of digestive system cancer.

Abbreviations: VOCs: Volatile organic compounds; DOR: diagnostic odds ratios.
