# Supplementary material for: Volatile organic compounds as a potential screening tool for neoplasm of the digestive system: a meta-analysis
Source: Sci Rep. 2021 Dec 9;11:23716. doi: 10.1038/s41598-021-02906-8 (PMC8660806; doi:10.1038/s41598-021-02906-8)
Supplement: Supplementary file 5 — Supplementary Figure S4. [file 41598_2021_2906_MOESM5_ESM.docx]

Supplemental figure 4. Forest plot of meta-regression and subgroup analyses of sensitivity and specificity VOCs in screening digestive system neoplasm.

Abbreviations: (a)Asian; (b)non-Asian (European and American); (c)total patients ≥100 cases; (d)total patients <100 cases; (e)healthy control; (f)non-cancer control. MS= mass spectrometry; IMS= ion mobility spectrometer; VOCs: Volatile organic compounds
